# Blueberry Extract as a Source of Natural Antioxidants to Improve Thermal Stability of Standard Sunflower Oil

**DOI:** 10.3390/antiox15050590

**Published:** 2026-05-07

**Authors:** Pamela Laera, Giacomo Squeo, Roccangelo Silletti, Michele Faccia, Francesco Caponio

**Affiliations:** Food Science and Technology Unit, Department of Soil Plant and Food Sciences, University of Bari “Aldo Moro”, Via Amendola 165/A, 70126 Bari, Italy; p.laera9@phd.uniba.it (P.L.); roccangelo.silletti@uniba.it (R.S.); michele.faccia@uniba.it (M.F.); francesco.caponio@uniba.it (F.C.)

**Keywords:** oxidative stability, natural extract, vegetable oil, industry applications, antioxidant

## Abstract

Vegetable oils are highly susceptible to thermo-oxidative degradation, leading to the deterioration of quality, the development of undesirable off-flavours, and the generation of potentially harmful compounds that may pose risks to human health. Although synthetic antioxidants are commonly used to mitigate these effects, growing safety concerns have stimulated research interest in natural alternatives. In the present study, a selection of commercially available plant extracts were carefully chosen and evaluated to assess their efficacy in enhancing the thermal oxidative stability of standard sunflower oil. Among the extracts tested, one blueberry extract (BE-B) exhibited the highest phenolic content as well as the strongest antioxidant activity. When incorporated into oil, BE-B significantly delayed the formation of primary oxidation products and the development of triacylglycerol polymers during heating, with the protective effects becoming more pronounced at higher concentrations. Volatile compound analysis further confirmed a substantial reduction in α,β-unsaturated aldehydes, which are recognized as major contributors to rancidity and oil degradation. Overall, these findings indicate that BE-B represents a highly promising natural antioxidant, capable of improving the quality and stability of vegetable oils, and underscore the potential for blueberry extract to be employed in commercial applications within the edible oil industry.

## 1. Introduction

Vegetable oils represent an essential component of the human diet and play a crucial role in the global agro-food industry. Their quality largely depends on oxidative stability, defined as the ability of the oil to resist oxidation during storage or thermal treatment [1]. Oxidative stability directly affects both the safety and sensory properties of the final product, and autoxidation is the primary mechanism of deterioration in oils rich in unsaturated fatty acids. This complex radical chain process involving initiation, propagation, chain branching and termination reactions is accelerated by pro-oxidant factors including oxygen, light, transition metals, and elevated temperatures [2]. Thermal treatments at high temperatures particularly promote the formation of secondary oxidation products, which may negatively affect oil quality and safety [3]. Oxidation hydroperoxides (primary oxidation products) and α,β-unsaturated aldehydes (secondary oxidation products) are key indicators of advanced oxidative deterioration and are considered potentially harmful [4]. In addition, extended heating leads to the formation of high-molecular-weight oxidized polymers, such as dimers, trimers, and oligomers of triglycerides, which are markers of severe thermal-oxidative degradation and reflect advanced quality deterioration [5]. To mitigate these effects, the addition of antioxidants is a widely adopted strategy [6].

Synthetic antioxidants, such as BHA and BHT, have historically been used to delay lipid oxidation [7]. However, concerns regarding their potential toxic and carcinogenic effects [8] have driven increasing scientific and industrial interest toward natural antioxidants, primarily derived from fruits, vegetables, or agro-industrial by-products [9]. Natural compounds such as phenolics, carotenoids, and tocopherols have been extensively investigated for their ability to slow oxidative reactions during thermal processing. Several studies have demonstrated that plant extracts rich in bioactive molecules can significantly enhance the thermal stability of vegetable oils. For instance, grape seed extracts showed higher protective effects than BHT in heated sunflower oil [10]; similarly, rosemary extracts exhibited strong antioxidant activity attributed to phenolic diterpenes such as carnosol and carnosic acid [11]. Further, spinach leaf extract increased thermal resistance due to its phenolic content [12], whereas carotenoids recovered from tomato residues improved oil stability depending on the extraction method employed [13].

Despite the extensive literature on natural antioxidants, the identification of novel plant extracts with potential application in edible oils remains an active area of research. A wide variety of plant matrices may represent promising sources of effective antioxidant compounds beyond the one most commonly used, including berries and cruciferous vegetables. In particular, blueberries are interesting for their high content of bioactive compounds [14], *Brassicaceae* due to their phytochemical profile, which includes polyphenols, glucosinolates, and other bioactive compounds [15], and blackcurrants (*Ribes nigrum* L.) as a rich source of anthocyanins, flavonols, phenolic acids, proanthocyanidins, and vitamin C [16].

However, it is well known that extracts obtained at lab scale directly from fresh plant material and/or by-products can exhibit considerable variability due to factors such as cultivar, geographic origin, agricultural practices, harvest season, and storage conditions. Furthermore, phytochemical yields can vary significantly depending on the extraction protocol, solvent system, and analytical method used, complicating the reproducibility and comparability of experimental results [17]. In contrast, commercial dry extracts offer greater batch-to-batch consistency, standardized phytochemical content, and greater stability. Accordingly, for testing and proposing the real industrial use of new natural extracts against the oxidative degradation of edible vegetable oils, different aspects must be considered, such as: (i) the commercial availability; (ii) antioxidant potential; (iii) reproducibility/consistency of the product.

Based on the above considerations, the objective of this study was to evaluate the antioxidant efficacy of four selected commercial extracts (a broccoli dry extract, two blueberry dry extracts, and a blackcurrant dry extract) in limiting the degradation process of sunflower oils subjected to thermo-oxidation. The formation of primary oxidation products, secondary volatile compounds, and advanced polymeric structures were investigated. The novelty of this study lies in evaluating the antioxidant performance of plant extracts such as broccoli, blueberry, and blackcurrant in lipid systems under thermo-oxidative conditions, which have not been extensively investigated despite their well-documented antioxidant properties. In addition, this study employs commercially available standardized plant extracts rather than laboratory-prepared extracts. While this does not represent a novelty per se, it allows for addressing important issues related to reproducibility, scalability, and industrial applicability that are often associated with lab-scale extraction procedures. This approach therefore provides a realistic assessment of the potential use of natural antioxidants in food industry applications.

## 2. Materials and Methods

Broccoli dry extract (BR), two blueberry dry extracts (BE-A and BE-B), and blackcurrant dry extract (RN) were purchased from Farmalabor SRL (Canosa di Puglia, Italy). Standard sunflower oil (Selex, Trezzano sul Naviglio, Italy), acquired from a local market, showed the following fatty acid composition: C_18:2_ (58.12%), C_18:1_ (30.89%), C_16:0_ (6.63%), C_18:0_ (3.59%), C_20:0_ (0.27%), C_18:3_ (0.22%), and C_16:1_ (0.12%).

Total phenolic content (TPC) was determined using the Folin–Ciocalteu method. In brief, 980 μL of Milli-Q water, 20 μL of appropriately diluted extract, and 100 μL of Folin–Ciocalteu reagent were mixed in a cuvette. After 3 min, 800 μL of 7.5% Na_2_CO_3_ solution was added, and samples were incubated in the dark for 60 min. Absorbance was measured at 720 nm (Agilent Technologies, Santa Clara, CA, USA). Results were expressed as mg gallic acid equivalents (GAEs) per gram of extract (mg GAE g^−1^). Antioxidant activity was evaluated using the ABTS assay. In brief, 50 μL of the diluted extract were mixed with 950 μL of the ABTS solution in a cuvette. After 8 min, the decrease in absorbance at 734 nm was recorded using a Cary 60 UV–Vis spectrophotometer (Agilent Technologies, Santa Clara, CA, USA). Results were expressed as μmol Trolox equivalents per gram (μmol TE g^−1^). All analyses were carried out in duplicate.

Sunflower oil was fortified with commercial extracts (broccoli, blueberry, blackcurrant) at concentrations of 0.3, 1, 5, 7.5 and 10 mg g^−1^. A minimal amount of distilled water (1 mL per 10 g of oil) was used to solubilize the extracts, and the mixture was homogenized using an Ultra-Turrax T-18 (IKA-Werke GmbH & Co. KG, Staufen, Germany). Oxidative stability under accelerated conditions was evaluated using the RapidOxy^®^ apparatus (Anton Paar, Blankenfelde-Mahlow, Germany), as described in [18]. Samples (1 ± 0.1 g) were incubated at 140 °C under an initial O_2_ pressure of 700 kPa (grade 5.0, SAPIO, Monza, Italy). The instrument monitored the decrease in oxygen pressure over time. The induction time (IT, min), defined as the time required for a 10% reduction in initial O_2_ pressure, was recorded as a measure of oil stability. The analysis was carried out in duplicate.

The extract showing the highest IT in the RapidOxy^®^ test was chosen for LC-MS characterization and further experimentation, as described in the following sections. Phenolic compounds in the blueberry extract BE-B were characterized by LC-MS in accordance with [19]. Analyses were performed on a UHPLC Ultimate 3000RS Dionex coupled to a linear ion trap mass spectrometer (LTQ Velospro, Thermo Fisher Scientific, Waltham, MA, USA) via a H-ESI II interface. Separation was achieved using a Hypersil GOLD aQ C18 column (100 × 2.1 mm, 1.9 μm) at 30 °C with a flow rate of 0.3 mL min^−1^. Mobile phases consisted of water with 0.1% formic acid (A) and acetonitrile with 0.1% formic acid (B), with a linear gradient from 98% to 30% A over 20 min, followed by 4 min isocratic at 30% A and 9 min re-equilibration. The PDA detector registered the spectrum from 220 to 600 nm. MS conditions were as follows: capillary temperature 320 °C, source temperature 280 °C, nebulizer gas N_2_, sheath gas flow 33 psi, auxiliary gas flow 5 arbitrary units, S-Lens RF 60%. Samples were analyzed in full-scan (from 100 to 1000 *m*/*z*) and data-dependent MS^2^ modes (200 to 1000 *m*/*z*; activation level of 500 counts; isolation width of 2 Da; CID energy of 35).

Fortified sunflower oil was heated in a ventilated oven at 180 °C for 120 min, with samples collected at 10, 20, 30, 60, and 120 min, following [10], with some modifications. Standard sunflower oil served as a control. Two fortification levels were tested: 1 mg g^−1^ and 10 mg g^−1^ of BE-B, resulting in 18 total samples. Two independent experimental batches were performed in order to evaluate both analytical repeatability and overall reproducibility, including sample preparation and thermal treatment. Sample placement in the oven was randomized to avoid systematic errors.

Peroxide value (PV) was determined according to [20]. To determine the amount of triacylglycerol oligopolymers (TAGPs), the oil samples were solubilized in tetrahydrofuran (THF) and analyzed by high-performance size-exclusion chromatography (HPSEC), employing THF as the mobile phase at a flow rate of 1 mL min^−1^. The HPSEC system consisted of a Series 200 pump (Perkin-Elmer, Shelton, CT, USA) equipped with a Rheodyne injector, a 50 µL loop, a PL-gel guard column (Perkin-Elmer, Beaconsfield, UK) measuring 5 cm × 7.5 mm i.d., and two PL-gel columns (Perkin-Elmer, Beaconsfield, UK), each 30 cm × 7.5 mm i.d. The columns were packed with highly cross-linked styrene–divinylbenzene copolymer containing 5 µm particles and a pore size of 500 Å. Detection was performed using a Series 200A refractive index detector (Perkin-Elmer). Peak identification and quantification were conducted as previously described [21]. The analysis of volatile compounds was performed using HS-SPME-GC-MS, following [22], with minor modifications. Briefly, approximately 1 g of sample was placed in a 20 mL vial. The vial was then sealed and conditioned at 40 °C for 2 min, after which the SPME fibre (50/30 µm DVB/CAR/PDMS; Supelco, Bellefonte, PA, USA) was exposed to the vial headspace for 20 min. Desorption was carried out directly in the GC-MS injector (Agilent 6850 series gas chromatograph coupled to an Agilent 5975 series mass spectrometer; Agilent Technologies, Santa Clara, CA, USA) at 250 °C for 2 min. Separation was achieved on a HP-Innovax polar column (60 × 0.25 mm, 0.25 μm film thickness). Volatile compounds were identified by comparison of their mass spectra with those in the NIST library, and only those showing a match quality above 70% were retained. The total ion count areas of the selected peaks were used for data elaboration. Analyses were performed in duplicate. All data are expressed as mean ± standard deviation. The coefficient of variation (CV%) between independent batches was also calculated to further assess experimental reproducibility; it was consistently below 10%, indicating good overall experimental reliability [23]. One-way analysis of variance (ANOVA), followed by Tukey’s Honestly Significant Differences (HSD) test for multiple comparisons at a significance level of α = 0.05, were used to highlight significant differences among the extract or the percentages. All statistical tests were performed using Minitab 19 Statistical Software (Minitab Inc., State College, PA, USA). Principal component analysis (PCA) was performed using CAT [24].

## 3. Results and Discussion

### 3.1. Chemical Characterization and Antioxidant Potential of Commercial Extracts

The commercial extracts were preliminarily characterized for their total phenolic content (TPC) and antioxidant activity (ABTS). The results are reported in Table 1.

As can be observed, significant differences in terms of both TPC and antioxidant activity emerged. The most interesting outcome was observed for the BE-B extract, which had a TPC value of about one order of magnitude greater than the other extracts considered in the study. The results of ABTS almost confirmed this trend. BE-A also showed very interesting results, ranking second in descending order among the extracts tested in terms of both TPC and ABTS. Finally, BR and RN had the lowest significant phenolic content, not significantly different among them, while, in terms of in vitro antioxidant activity, RN performed better than BR. In comparison with previous reports, BR showed a TPC considerably higher than the 6.38 mg GAE g^−1^ reported by [25], as well as RN, which exceeded the value of 12.8 mg GAE g^−1^ reported by [26]. Overall, both blueberry extracts produced promising results, although BE-B stood out against BE-A. This is in accordance with the technical sheet of the extracts.

The extracts were then added to sunflower oil at different increasing concentrations, and the samples subjected to an accelerated oxidation test. The results, expressed as induction time, are reported in Table 2.

The induction time of the control sunflower oil (CO) was 33.86 min. The addition of BR, RN, or BE-A did not improve the oxidative stability of sunflower oil. In contrast, BE-B showed a marked effect: at a concentration of 1 mg g^−1^, the induction time increased to 38.40 min, and at 10 mg g^−1^ it reached 48.51 min, corresponding to an increase of 4.54 min and 14.65 min, respectively, compared to the control. The capability of natural extracts to enhance oxidative stability under accelerated conditions is known [27] and commonly attributed to the high content in phenolic compounds, which may act as effective radical scavengers. Nonetheless, the results in Table 2 show how such activity in real conditions (i.e., in oil) might depend on the specific product. It is worth recalling that natural extracts, like those tested in this study, are rich in hydrophilic reducing compounds, whose solubility, and consequent activity, in oil can be strongly reduced [28]. Overall, promising results were obtained for BE-B that, even at low concentrations, provide measurable antioxidant protection, with the effect being concentration-dependent.

Based on this preliminary screening, BE-B was selected for further investigations which, first of all, consisted of phenolic characterization. Table 3 shows the phenolic composition of BE-B. Twelve compounds were tentatively identified according to the literature [19,29].

Compounds identified include chlorogenic acid, anthocyanins such as delphinidin-3-O-glucoside and cyanidine-3-O-glucoside, flavonols, like kaempferol and myricetin, as well as other phenolic acids and glycosides. Such a profile was expected as blueberries are recognized as a rich source of bioactive phytochemicals, particularly flavonoids and anthocyanins, which are responsible for the vivid colours of the fruits and have been associated with various health benefits [30].

### 3.2. Effect of Thermal Treatment on Sunflower Oil Fortified with Blueberry Extract

**Figure 1:**
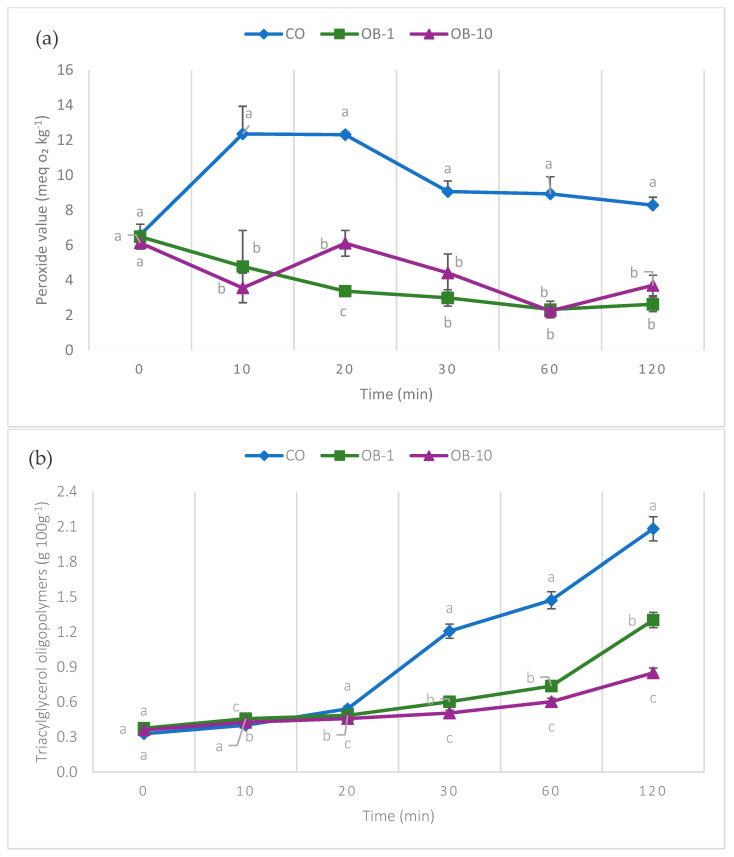
Effect of blueberry extract (BE-B) on oxidative degradation of sunflower oil during thermal treatment at 180 °C. (**a**) Peroxide value (meq O_2_ kg^−1^) evolution over time (min). (**b**) Evolution of triacylglycerol oligopolymers (g 100 g^−1^) over time (min). CO: standard sunflower oil; OB-1: standard sunflower oil with 1 mg g^−1^ blueberry extract; OB-10: standard sunflower oil with 10 mg g^−1^ blueberry extract. Data are expressed as mean ± standard deviation (*n* = 2). Different letters at the same time point indicate statistically significant differences among samples, as determined by one-way ANOVA followed by Tukey’s post hoc test (*p* < 0.05).

The BE-B extract was added to sunflower oil in two different concentrations (1 mg g^−1^ and 10 mg g^−1^) and subjected to an oven thermal treatment at 180 °C. Figure 1a shows the evolution of the peroxide value during the oven test.

PV is an indicator of primary oxidation, reflecting the development of hydroperoxides during the initial stages of lipid oxidation [1]. In the CO, peroxide values increased rapidly during the initial phase of heating and remained at relatively high levels throughout the thermal treatment. In contrast, oils fortified with the blueberry extract (OB-1 and OB-10) exhibited lower PV over the entire heating period. In these samples, PV remained approximately constant, oscillating around values close to 3–4 meq O_2_ kg^−1^. Despite minor fluctuations, the consistently lower PV observed in enriched oils compared to the control confirms the protective effect of the blueberry extract against primary lipid oxidation. Statistical analysis showed significant differences among treatments at most time points, supporting the enhanced oxidative stability of extract-enriched oils. These findings are consistent with previous studies reporting that phenolic-rich plant extracts reduce hydroperoxide formation and improve the thermal stability of vegetable oils [31]. However, it is noteworthy to recall that such an effect has not been previously reported during an oven test in standard sunflower oil. The results presented confirm that, although basically rich in hydrophilic antioxidants, BE extract can effectively be used for oil thermo-oxidative stability.

Moving to triacylglycerol oligopolymers, Figure 1b shows their evolution during heating. In CO, TAGP gradually increased over time, reaching values close to 2 after 60 and 120 min. Oils fortified with blueberry extract, at both 1 mg g^−1^ and 10 mg g^−1^, underwent lower TAGP formation throughout the heating period, with the 10 mg g^−1^ treatment showing the most pronounced effect. The difference between the control and the treated samples became more apparent over time, indicating that the extract contributed to limiting TAGP formation during the heating process.

The observed dose-dependent effect (10 mg g^−1^ > 1 mg g^−1^) suggests that higher concentrations of phenolics enhance this protective action, which persists over the 120 min heating period. The effect likely involves radical scavenging by phenolics, which can donate hydrogen or electrons to lipid radicals and thus slow the formation of cross-linked polymers [1]. Similar trends have been reported in studies where natural antioxidants reduced the accumulation of polymeric (such as TAGP) and polar compounds in heated oils [2,14]. Polar compounds arising from lipid oxidation are associated with negative impacts on oil viscosity, clarity, and flavour, and may include potentially harmful high-molecular-weight products. By limiting their formation, the oil maintains a more stable chemical–physical profile and reduces the risk of secondary (potentially toxic) degradation products, which is a key advantage for cooking applications or industrial processing. The effect is analogous to what has been shown when adding other natural antioxidative extracts: for example, enrichment of sunflower oil with phenolic extracts from olive-leaf juice markedly suppressed the increase in both total polar compounds and polymer content during heating cycles compared with non-enriched oil [32]. Moreover, studies on different vegetable oils have documented that thermal and frying stresses lead to progressive accumulation of various degradation products, including oxidized triglyceride monomers (OxTGs), triglyceride dimers (TGDs), and oligomers, whose formation kinetics strongly depend on temperature, degree of unsaturation, and the presence or absence of antioxidants [33]. In some cases, oils enriched with natural or synthetic antioxidants showed delayed accumulation of polar and polymeric products, confirming that antioxidants can extend the usable lifetime of frying oils [34]. Given these considerations, the data from the present study support the potential of blueberry-derived phenolic extract as a natural antioxidant to enhance the thermal stability of sunflower oil.

The autoxidation, and strictly related thermo-oxidation, are per se not static but evolving processes. Hence, with the aim of gaining a global overview, different complementary parameters should be considered. Together with those already presented, volatile compounds and secondary lipid oxidation products are very informative markers and are primarily responsible for the negative sensory characteristics of oxidated oils [1,35]. The peak area of key aldehydes after 120 min of thermal treatment decreased markedly and, in line with the reduced formation of both primary oxidation products (PVs) and triacylglycerol oligopolymers, the volatile profile revealed clear differences between CO, OB-1 and OB-10. In further detail, (Z)-2-heptenal decreased from 12.59% in the control to 3.98% in OB-1 and was not detected in OB-10. (E)-2-octenal decreased from 0.96% to 0.56% and was not detected in OB-10, while (E,E)-2,4-decadienal decreased from 3.98% to 0.25% and 0.38% in OB-1 and OB-10, respectively.

**Figure 2:**
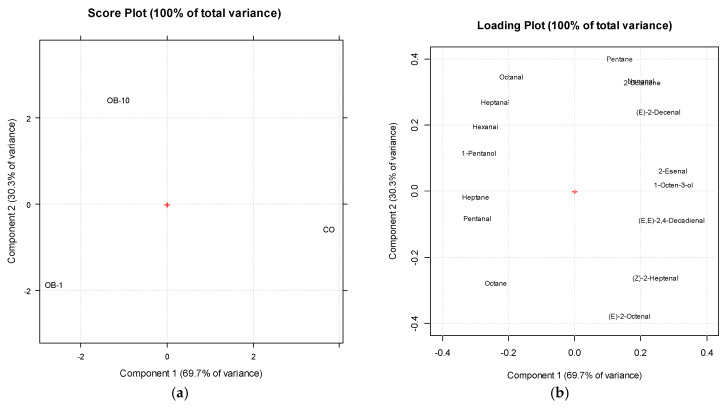
Score (**a**) and loading (**b**) plots from PCA of volatile compounds in sunflower oil samples after 120 min of thermal treatment at 180 °C.

Figure 2 shows the results of the PCA carried out on volatile data after 120 min of thermal treatment.

The first two principal components (PC1 and PC2) explain, respectively, 69.7% and 30.3% of the total variance. From the score plot (Figure 2a), a clear clustering of the samples can be easily observed. Along PC1, the control oil and those added with BE separate. The corresponding loading plot shows that compounds like (E)-2-octenal, (Z)-2-heptenal, (E,E)-2,4-decadienal, pentanal, and nonanal, typically associated with lipid degradation during heating and oil rancidity [36], mostly characterize CO. α,β-unsaturated aldehydes such as (E,E)-2,4-decadienal, (E)-2-octenal, and (Z)-2-heptenal are characterized by a very low odour threshold and are major contributors to rancid and fried off-flavours in oxidized oils [37]. Their strong reduction or complete absence in blueberry-enriched oils confirms previous findings, suggesting a marked delay in lipid oxidation, which might translate into a rancid odour perception by consumers. Therefore, the modulation of the volatile profile by blueberry extracts likely results in a reduced sensory perception of thermo-oxidative deterioration.

The observed changes in the volatile profile suggest that the blueberry extract affected the oxidation process, leading to a slower or altered progression of lipid degradation. The most remarkable changes concern α, β-unsaturated aldehydes, which are key indicators of advanced thermo-oxidative degradation [38]. Compounds such as (Z)-2-heptenal, (E)-2-octenal, and (E,E)-2,4-decadienal were reduced in the presence of the extract, and in OB-10 some were undetectable. These aldehydes arise from secondary β-scission of linoleate hydroperoxides and are typically abundant in severely oxidized oils [39]. This finding aligns with previous studies showing that polyphenol-rich extracts reduce the formation of α, β-unsaturated aldehydes and other toxic secondary compounds during heating or frying of vegetable oils [40].

## 4. Conclusions

In conclusion, the results of this study demonstrate the potential of blueberry extract to enhance the oxidative stability of sunflower oil under thermal treatment. Fortification with the extract significantly reduced the formation of peroxides and polymeric compounds, providing protection during the initial stages of lipid oxidation as well as under prolonged heating. This protective effect was further supported by the lower levels of secondary oxidation products, including α,β-unsaturated aldehydes, indicating that the extract slows the progression toward advanced thermo-oxidative degradation. The observed dose-dependent response highlights the importance of sufficient phenolic content to maintain stability under high-temperature exposure. Overall, these findings suggest that blueberry-derived extracts represent a promising natural alternative to synthetic antioxidants for improving the quality and safety of vegetable oils. From a broader perspective, they also underscore opportunities for enhancing blueberry by-products as sources of functional antioxidants, aligning with sustainability goals. Although the present study demonstrates the promising antioxidant activity of blueberry extract under accelerated thermal conditions, it should be noted that sensory attributes (such as colour and flavour) as well as long-term storage stability were not evaluated. These aspects are critical for industrial application in edible oils, particularly when using coloured plant extracts. Moreover, the behaviour of hydrophilic phenolic compounds in lipid matrices remains complex, and factors such as limited solubility, phase distribution, and potential thermal degradation were not specifically investigated in the present work. Therefore, these aspects represent important limitations that should be considered when interpreting the observed antioxidant effects. Future studies could also explore strategies to enhance the incorporation of hydrophilic extracts into lipid matrices, for instance through improved solubilization or formulation approaches, as well as evaluate their performance in real food systems under industrial processing conditions.

## Figures and Tables

**Table 1:** Total phenolic content and antioxidant activity of the commercial extracts.

Extract	TPC (mg GAE g^−1^)	ABTS (mg TE g^−1^)
BR	25.30 ± 1.12 c	19.88 ± 0.85 d
BE-A	55.63 ± 1.36 b	151.00 ± 4.60 b
BE-B	413.91 ± 1.32 a	339.65 ± 0.68 a
RN	21.56 ± 1.81 c	46.84 ± 1.92 c

Data are presented as mean ± standard deviation (*n* = 2). Different letters indicate statistically significant differences, as determined by one-way ANOVA followed by Tukey’s post hoc test (*p* < 0.05). BR, broccoli dry extract; BE-A, blueberry dry extract A; BE-B, blueberry dry extract B; RN, blackcurrant dry extract.

**Table 2:** Induction time (min) under accelerated oxidation conditions (140 °C) of sunflower oil added with different concentration of the commercial dry extracts.

Concentration	BR	BE-A	BE-B	RN
0.3 mg g^−1^	33.10 ± 0.73 a	33.43 ± 0.36 a	33.71 ± 0.01 c	33.43 ± 0.31 a
1 mg g^−1^	33.45 ± 0.34 a	33.77 ± 0.01 a	38.40 ± 0.37 b	33.36 ± 0.49 a
5 mg g^−1^	33.83 ± 0.07 a	33.54 ± 0.43 a	38.89 ± 0.18 b	33.33 ± 0.59 a
7.5 mg g^−1^	33.99 ± 0.20 a	33.81 ± 0.23 a	37.38 ± 0.64 b	33.75 ± 0.09 a
10 mg g^−1^	34.17 ± 0.35 a	34.08 ± 0.04 a	48.51 ± 0.71 a	33.94 ± 0.08 a

Data are presented as mean ± standard deviation (*n* = 2). Different letters within the same column indicate statistically significant differences, as determined by one-way ANOVA followed by Tukey’s post hoc test (*p* < 0.05). BR, broccoli dry extract; BE-A, blueberry dry extract A; BE-B, blueberry dry extract B; RN, blackcurrant dry extract.

**Table 3:** LC-MS phenolic profile of blueberry extract (BE-B).

Compounds	RT (min)	[M+H]+ (*m*/*z*)	MS/MS (*m*/*z*)
3-caffeoylquinic acid	4.39	353.30	191.08
Delphinidin-3-O-glucoside	5.88	465.21	285.02
Cyanidine 3-O-glucoside	6.38	447.22	284.99
Malvidin-3-O-glucoside	6.68	495.25	315.02
Kaempferol-3-O-xyloside	6.92	417.30	283.00
Myricetin	7.63	319.31	164.96
Petunidine-3-O-glucoside	8.04	479.25	299.00
Kaempferol	8.20	284.30	102.05
Delphinidin-3-(6″-acetylglucoside)	8.64	509.17	329.07
Malvidin-3-(6″-acetylglucoside)	8.87	535.17	371.10
Isoramnetin 3-glucoside	9.95	477.19	300.98
Glucose cinnamoyl	37.35	311.47	183.01

## Data Availability

All data generated or analyzed during this study are included in the article. Further inquiries can be directed to the corresponding author.
